# Hepatic steatosis, a lesion reported in captive aged common marmosets

**DOI:** 10.31491/apt.2021.03.052

**Published:** 2021

**Authors:** Olga Lucia Franco-Mahecha, Sebastian E. Carrasco

**Affiliations:** a Division of Comparative Medicine, Massachusetts Institute of Technology, Cambridge, MA 02139, USA.

**Keywords:** *Callithrix jacchus*, marmoset, hepatic steatosis, non-alcoholic fatty liver disease, lipid vacuoles

## Abstract

Hepatic steatosis, also known as fatty liver, is a spontaneous lesion caused by the abnormal accumulation of triglycerides within hepatocytes that has been described in different laboratory-housed nonhuman primate species. Aging is considered a risk factor in the progression of this lesion in humans and captive rhesus macaques. Hepatic steatosis has been reported in sexually mature adult and aged-adult captive common marmosets. Macroscopic changes in the liver may be evident in advanced stages of this condition and are characterized by hepatomegaly with multifocal to coalescing to regionally extensive pale-tan to yellow, soft foci throughout the hepatic lobes. Biochemical abnormalities in these cases include significantly increased levels in triglycerides, insulin, and γ-glutamyltransferase (GGT). Definitive diagnosis is by histopathology and demonstration of lipid accumulation within hepatocytes. Histopathology is characterized by large coalescing areas of periacinar to periportal microvesicular steatosis mixed with clusters of macrovesicular steatosis, and variable degrees of lobular inflammation. Vacuolated hepatocytes containing intracytoplasmic lipid material is demonstrated by positive staining to Sudan IV and/or Oil red-O.

Hepatic steatosis, also known as fatty liver, is a spontaneous lesion in laboratory-housed nonhuman primates experiencing obesity and other related metabolic diseases [1–3]. It is characterized by an abnormal accumulation of triglycerides within the cytoplasm of hepatocytes and considered the first lesion seen in cases with non-alcoholic fatty liver disease (NAFLD) [4]. This condition is prevalent in the elderly human population and captive aged non-human primates, such as rhesus macaques (*Macaca mulata*) and bonnet macaques (*Macaca radiata*) [2,5–7]. NAFLD also occurs spontaneously or is induced experimentally, by dietary changes, in other captive nonhuman primate species, such as common marmosets (*Callithrix jacchus*), cynomolgus macaques (*Macaca fascicularis*), baboons (*Papio hamadryas sp*.), and vervet monkeys (*Chlorocebus pygerythrus*) [3,8]. In our diagnostic pathology service, hepatic steatosis is often noted in necropsied adult and adult-aged marmosets with or without other comorbidities (S. E. Carrasco, Personal communication, March, 2021). On gross examination, the livers of marmosets with steatosis are enlarged exhibiting multifocal to regionally extensive areas of pale-tan to yellow discoloration that are soft to friable on sectioning. Serum bio-chemistry is characterized by increased levels in triglycerides, γ-glutamyltransferase (GGT), and insulin [8]. The case shown in Figure 1 exhibited a progressive elevation in GGT and Alkaline phosphatase (ALP). Histopathology along with special stains for lipid content in liver samples is considered the gold standard in the diagnosis of hepatic steatosis. Steatosis is often noted in centrilobular and mid-zonal hepatocytes but can also extend to periportal regions (Figure 1A). Hepatocytes are moderately to markedly enlarged by well-defined, variably sized intracytoplasmic clear vacuoles that flatten and displace the nuclei to the periphery (Figure 1B and 1C). Within the affected areas, there are swollen hepatocytes with small intracytoplasmic vacuoles and centrally placed indented nuclei (Figure 1B). Lipid accumulation in hepatocytes is confirmed by positive Oil Red O staining (Figure 1D). In severe cases, hepatic steatosis can progress to Nonalcoholic steatohepatitis (NASH) in which hepatic steatosis is associated with areas of lymphocytic and histiocytic inflammation, pericellular fibrosis, and ballooning degeneration of hepatocytes [8]. Masson trichrome or Picrosirius red stains can be used to evaluate the degree of fibrosis in these cases. The presence of intracytoplasmic keratin-rich, hyaline inclusions (Mallory-Denk bodies) in ballooned degenerating hepatocytes can be demonstrated by immunohistochemistry for cytokeratin 8/18, p62, and ubiquitin [9]. Mallory-Denk bodies are made up of intermediate cytokeratin 8/18 filament proteins that are ubiquitinated or bound by scaf-folding proteins, such as p62/Sequestosome-1 (p62) [9]. NAFLD is reported mainly in middle-aged and elderly human population and has become the second leading cause of liver transplantation in the United States [7]. Elderly people with chronic conditions such as visceral obesity, diabetes, insulin resistance, hypertension and/or dyslipidemia are at higher risk of developing NAFLD/NASH, cirrhosis, and hepatocellular carcinoma (HCC) [7]. The pathogenesis of NAFLD in humans involves a complex interaction among hormonal, genetic, nutritional, and intestinal microbiome factors [4]. Previous studies delineating mechanisms involved in NAFLD have largely used rodent models (for details on pathophysiological mechanisms of NAFLD/NASH see [11,12]). Old world non-human primates, such as rhesus macaques [5] and bonnet macaques [6] have been proposed as animal models of NAFLD/NASH in aging studies. Hepatic lesions in rhesus macaques diagnosed with NAFLD display large regions of micro- and macrovesicular steatosis [5]. Although perisinusoidal and periportal fibrosis, lobular inflammation, and ballooning hepatocytes are noted in steatotic livers, these histological changes are not always observed in this model of NAFLD/NASH in aged rhesus macaques [5]. Aged rhesus macaques (older than 15-year-old) diagnosed with metabolic syndrome (defined by the presence of a cluster of metabolic factors, such as obesity, hyperglycemia, hypertriglyceridemia, hypercholesterolemia, and high blood pressure) have a higher risk of developing hepatic steatosis [5]. Aged bonnet macaques (18–20-year-old) exhibit hepatic lesions similar to those histopathological changes seen in humans with NAFLD [6]. The common marmoset has gained interest in the past decade as a powerful model for aging research studies [1]. Marmosets have shorter lifespans (approximately 10–15 years), produce larger viable litter-sizes per delivery, and are easier to handle and maintain than traditional Old-World monkeys used in biomedical research. These characteristics place the common marmoset as an attractive NHP model to study pathological changes associated with aging. For example, common marmosets can serve as a model for age-related neurodegenerative/cognitive disorders, chronic renal diseases, and age-related cardiovascular and metabolic disturbances associated with obesity [1,10]. Recently, captive common marmosets were described as an attractive model of NAFLD/NASH as it recapitulates several clinicopathological aspects of this condition in humans. Hepatic steatosis was primarily described in captive marmosets with evidence of obesity and insulin resistance and occurred in sexually mature adult and aged-adult captive marmosets [8]. Marmosets with hepatomegaly also had higher body condition scores and higher body and fat mass than marmosets without hepatomegaly [8]. Histological changes in the liver of these animals showed diffuse to multifocal regions of micro- and macrovesicular steatosis and variable degrees of lobular inflammation and pericellular fibrosis [8]; findings comparable to lesions observed in other NHP models of NAFLD. Ballooning hepatocellular degeneration and Mallory-Denk bodies are also observed in severe cases of NASH in marmosets [8].

## Figures and Tables

**Figure 1.**
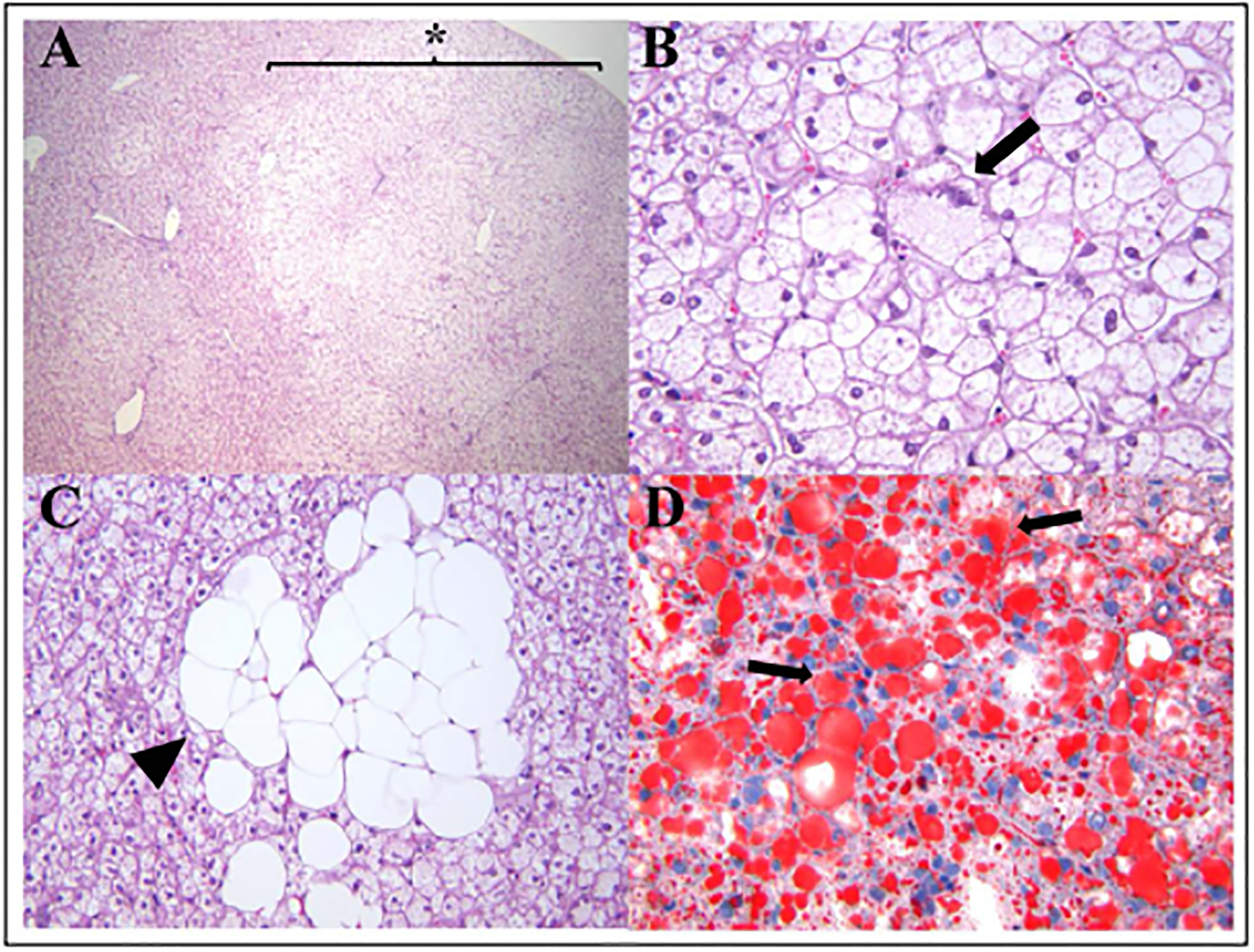
Histological changes associated with hepatic steatosis in a 14-year-old female marmoset. **(A)** Sections of liver have multifocal large regions of microvesicular steatosis extending from periacinar to periportal regions (star, 40x). **(B)** Areas of microvesicular steatosis are characterized by swollen hepatocytes containing small clear intracytoplasmic vacuoles and eccentrically displaced nuclei (arrow, 400x). **(C)** Higher magnification of macrovesicular steatosis demonstrating clusters of hepatocytes with large intracytoplasmic lipid vacuoles completely effacing the nuclei (arrowhead; 400x). **(D)** Lipid content in areas of microvesicular and macrovesicular hepatic steatosis is demonstrated by Oil red-O staining of hepatocytes (red, arrows; 400x).
